# Stigma and chronic illness: A comparative study of people living with HIV and/or AIDS and people living with hypertension in Limpopo Province, South Africa

**DOI:** 10.4102/curationis.v41i1.1879

**Published:** 2018-10-25

**Authors:** Erhabor S. Idemudia, Matthew O. Olasupo, Mantwa W. Modibo

**Affiliations:** 1 Population and Health Research Entity, Faculty of Humanities, North-West University, South Africa

## Abstract

**Background:**

Stigma among people with chronic illnesses exists, preventing many sufferers from presenting for treatment especially in South Africa.

**Objectives:**

This study compared stigma experiences of people living with human immunodeficiency virus and/or acquired immunodeficiency syndrome (HIV and/or AIDS) (PLWHA) and people living with hypertension (PLWHPT) in Limpopo Province of South Africa.

**Method:**

Using a cross-sectional design, 600 participants (300 PLWHA with mean age of 31 years, standard deviation of 8.2; and 300 PLWHPT with age of 55 years, standard deviation of 8.1) were purposefully sampled at HIV and/or AIDS and hypertension outpatient clinics. The perceived stigma of AIDS scale was used to assess stigma in the HIV and/or AIDS sample while the adapted version was used to assess stigma in PLWHPT. Data were analysed using independent *t*-test.

**Results:**

Results indicated that PLWHA experienced significantly higher enacted stigma (*t*(598) = −11.79, *p* < 0.001) as compared to PLWHPT. However, PLWHPT experienced significantly higher internalised stigma (*t*(598) = 37.56, *p* < 0.001) and perceived stigma (*t*(598) = 41.71, *p* < 0.001) than PLWHA.

**Conclusion:**

Stigma among people with chronic illnesses is existent. The stigma type is, however, dependent on the nature of the illness. Stigma reduction interventions among these populations are indicated.

## Introduction

Human immunodeficiency virus and/or acquired immunodeficiency syndrome (HIV and/or AIDS) is documented as supremely stigmatised (Tsai et al. 2017) and viewed more negatively relative to other chronic illnesses (Daftary 2012; Deribew et al. 2010; Idemudia & Matamela 2012). As a result of the cognisance of stigma and its adverse effects on the sufferers, government health authorities across the globe, South Africa inclusive and other relevant stakeholders have vigorously embarked on anti-stigma programmes and campaigns to date. Therefore, it is imperative to monitor and track stigma experiences among this population in response to the effectiveness or otherwise of the anti-stigma programmes.

Because hypertension may not come with symptoms, it makes it less stigmatised compared to HIV and/or AIDS, yet it is being regarded as one of the most prevalent chronic conditions and one of the prominent causes of death globally (WHO 2013), including South Africa (Ramaano et al. 2014). It is also known as a major risk factor for cardiovascular diseases (Kretchy, Owusu-Daaku & Danquah 2014) with debilitating effects (Ivarsson, Ekmehag & Sjöberg 2015). Despite this fact, studies on stigma among this population are relatively sparse, both globally and in South Africa. Hence, the current study seeks to assess stigma experiences among a population of people living with HIV and/or AIDS (PLWHA) compared to people living with hypertension (PLWHPT).

People suffering from chronic illnesses present with behavioural deviations from what other people expect in society and social interactions, which are precursors of stigma. Once an individual is pronounced or labelled ill, a sense of stigma is induced. Essentially, the mere term ‘illness’ induces a sense of stigma (Beatty 2018; Pettit 2008). Variations in the definition of the concept of stigma have not been objected to because of the complex nature of the phenomenon and the multidisciplinary involvement of sociologist, psychologist, social workers and others (Beatty 2018; Pachankis et al. 2018). In the current study, stigma is defined from Goffman’s point of view, who defined it as an attribute that is deeply discrediting, an aspect of the self that is socially devalued, that reduces the bearer from a whole and usual person to a tainted, discounted one ( Pachankis et al. 2018).

Stigma has also been recognised to manifest through two mechanisms: instrumental and symbolic stigma (Darlington & Hutson 2017; Nyblade et al. 2018). Instrumental stigma involves stigma that arises as a result of a perceived threat to an individual’s well-being that ultimately brings about a negative attitude towards an individual perceived to be the threat (Perloff 2001). With reference to illness, people avoid or are hesitant to associate themselves with an individual considered ill as they are fearful that they will contract the disease. Symbolic stigma represents cognitive representation of people with certain cognitive schemata. That is, symbolic stigma manifests secondary to the association made between the stigmatised individual with negative attitudes towards the associated stigmatised symbol (Nyblade et al. 2018; Turan et al. 2017). For example, in relation to hypertension, people may associate hypertension with an unhealthy lifestyle. While in relation to HIV and AIDS, people may associate HIV and/or AIDS with the already prejudiced groups who are associated with moral transgressions and decadence, homosexuality, promiscuity, etc. Thus, the stigmatising ill people represent the prejudices towards the symbol associated with the disease.

Within the two broad stigma mechanisms, that is, instrumental and symbolic, stigma is recognised to occur through at least three processes, which are internalised, enacted and perceived stigma. Internalised stigma is conceptualised as a state in which the negative attributes and beliefs about the illness are permitted and accepted internally by the sufferer. Enacted stigma is a state in which an individual experiences prejudice and/or discrimination arising from others, while perceived stigma is conceptualised as a state in which an individual expects to experience stigma enactments (Rueda et al. 2012).

Furthermore, the illness aetiology plays a major role in the stigmatisation of that particular illness. Pre-existing stereotypes and prejudices have been recognised to influence stigmatising attitudes towards an illness (Pachankis et al. 2018). In relation to the present study, HIV and/or AIDS is often perceived as or associated with moral concepts of blame, responsibility and deservedness (UNAIDS 2013), resulting in PLWHA often being blamed as having brought the disease upon themselves by engaging in socially, culturally or morally prohibited or condemned behaviours (Fielden, Chapman & Cadell 2011; Patel et al. 2012). This is because morality is fundamental to African principles (Idemudia 2003) which make HIV and/or AIDS a relatively highly stigmatised medical condition (Daftary 2012; Idemudia & Matamela 2012). The association made between HIV and/or AIDS and sex, drugs, homosexuality, contagion and an unpleasant form of death makes it a powerfully stigmatised illness (DeMarco & Cao 2015; Mukoloa et al. 2014). Thus, HIV and/or AIDS is highly stigmatised owing to negative societal preconceptions allied with the negative associated behaviours.

According to Attribution Theory’s postulation (Heider 1958), an individual’s situation where causal attributions of controllability are tied to the stigmatised individual tend to elicit greater prejudice towards that individual. That is, HIV and/or AIDS is primarily perceived to emanate from internal, personal and morally wrong controllable acts. These cognitive connections and the attributions made about PLWHA as a result trigger different negative affective reactions, including stigma. With regard to hypertension, the increasing prevalence of the condition is often attributed to and/or blamed on lifestyle and dietetic factors, related to physical inactivity, alcohol and tobacco use, and high-sodium dietary intake (usually from processed and fatty foods) (Lloyd-Sherlock, Ebrahim & Grosskurth 2014). As a result, such societal preconceptions are the precursors of stigma. Despite that, not many comparative studies of HIV and/or AIDS, and other chronic medical conditions such as living with hypertension on experiences of stigma have been conducted, especially in South Africa. Therefore, the present study seeks to compare stigma experiences among PLWHA and PLWHPT in Limpopo Province of South Africa

## Methods

### Study design and setting

A cross-sectional design was used. Participants were recruited from three hypertension and HIV and/or AIDS outpatient clinics (hospital names withheld) in Capricorn District. Capricorn is one of the five districts in Limpopo Province. Other districts are Waterberg, Vhembe, Mopani and Greater Sekhukhune. This district was chosen because of its wide racial groups and high socio-economic standing compared to the other four districts.

### Participants and sampling technique

The study consisted of 600 participants, 300 PLWHPT and 300 PLWHA. A priori power analysis with G*Power was used to determine appropriate sample sizes for the two groups (N1 = N2) with an indication of generating significant results. The effect size (Cohen’s *d*) was set at 0.5 (medium effect) and Power (1-β err prob) set at 0.95. The demographic characteristics of participants are presented in Table 1. Because of the nature of the study participants, convenient sampling method was employed. Convenient sampling is a non-probabilistic sampling technique concerned with recruiting specified types of people because they are readily available or they are close at hand. Thus, patients with characteristics of interest (HIV and/or AIDS or hypertension) who were available and accessible at the time of data collection were sampled.

**TABLE 1:** Demographic characteristics of participants (*n* = 300).

Characteristics	PLWHA	PLWHPT
Age range	20–54 years	36–77 years
**Mean age (*SD*)**	**31 (8.2)**	**55 (8.10)**
**Gender *n* (%)**		
Male	125 (41.5)	141 (47.0)
Female	172 (57.1)	159 (53.0)
Missing value	3 (1.4)	-
**Marital status *n* (%)**		
Married	89 (29.6)	158 (52.7)
Never married	169 (56.1)	99 (33.0)
Divorced	6 (2.0)	14 (4.7)
Widowed	18 (6.0)	23 (7.7)
Missing value	18 (6.3)	6 (20.0)
**Educational level *n* (%)**		
Less than Grade 12	35 (11.6)	131 (43.7)
Grade 12	76 (25.2)	102 (34.0)
Tertiary education	164 (54.5)	61 (20.3)
Missing value	25 (9.5)	6 (2.0)
**Duration of diagnosis, *n* (%)**		
Less than 1 year	143 (47.5)	33 (9.3)
2–4 years	139 (46.5)	154 (51.3)
More than 5 years	18 (6.0)	113 (37.7)

*n*, number of participants; PLWHA, people living with HIV and/or AIDS; PLWHPT, people living with hypertension; *SD*, standard deviation.

### Research instrument

A paper-and-pencil questionnaire was used to collect data from the study participants. Section A of the questionnaire asked questions on personal information like age, gender, marital status, educational level and duration of diagnosis. The second section consisted of a standardised psychological scale, the perceived stigma of AIDS developed by Westbrook and Bauman (1996), which was used to assess perceived stigma in respondents. It is a 20-item, Likert-type scale that measures internalised, perceived and enacted stigma. Sample items include ‘I feel ashamed of having this illness’, ‘People with HIV are bewitched’, etc. The original scale was used to measure perceived stigma in PLWHA while the adapted version was used to assess perceived stigma in PLWHPT. Sample items in the adapted version for PLWHPT include ‘I feel ashamed of having this illness’, ‘People with hypertension are bewitched’, etc. Idemudia and Matamela (2012) had previously determined the suitability of using the original scale on South African population and found it reliable with a Cronbach’s alpha of 0.88. The authors conducted a pilot study consisting of 30 persons on the adapted version for PLWHPT and found it reliable with a Cronbach’s alpha of 0.83.

### Data analysis

G*Power software was used to determine the appropriate sample (N1 = N2) sizes for the study, given a predetermined effect size and power statistics. Data collected were analysed with SPSS (version 24). The scale comprised items that assess internalised, perceived and enacted stigma. The scale had 20 items that assessed internalised stigma, 20 items that assessed perceived stigma and 9 items that assessed enacted stigma. The participants had to respond on a 4-point Likert scale, with 1 indicating strongly agree and 4 indicating strongly disagree. With regard to enacted stigma, the participants had to respond on a 3-point Likert scale with 1 indicating no experience and 3 indicating a lot of experience. There were items that needed to be reversed. For internalised stigma, the following items were reversed: Items 10, 12, 17, 18, 19, 25 and 26. For perceived stigma, the reversed items were Items 30, 32, 37, 38, 39, 45. For enacted stigma items, no reverse was required. High scores were indicative of high stigma experience on each of the sub-scales. Descriptive statistics were calculated for the demographic characteristics. To determine group differences (PLWHA and PLWHPT) in terms of stigma experiences, independent *t*-test was employed.

### Ethical considerations

The North-West University ethics committee (ethical clearance no: NWU-00130-11-A9) and the ethics committee of Limpopo Provincial Department of Health (Ref: 4/2/2) duly approved the study. In addition, participants were made to fill an informed consent form and were assured of the confidentiality of their participation. The rights of not to participate and to withdraw from the study at any time were also guaranteed. The approval letter by the North-West University ethics committee was part of the documents submitted to the ethics committee of Limpopo Provincial Department of Health, while the approval letter by the ethics committee of Limpopo Provincial Department of Health was used to gain access to the study sites (three clinics where data were collected). Inclusion criteria included being diagnosed with HIV (for PLWHA) and being diagnosed with hypertension (for PLWHPT). All participants were aged 18 years and above. This was followed to ensure that all participants were able to give informed consent on their own. Those with multiple chronic conditions that may interfere with study outcomes were excluded from the study. The questionnaire was administered in English while interpretation was done for those who required translation. Data collection took approximately 6 weeks.

## Results

The test results (see Table 2) showed that PLWHA significantly experienced higher enacted stigma than PLWHPT (*t* = −11.79, *P* < 0.001). People living with hypertension relatively experienced higher internalised stigma than PLWHA over other stigma dimensions ($\bar{\text{x}}$ = 64.60, *SD* = 2.81 vs $\bar{\text{x}}$ = 44.53, *SD* = 8.82). This difference was statistically significant: *t* (598) = 37.56, *p* < 0.001. Perceived stigma was also significantly higher among PLWHPT relative to PLWHA: *t* (598) = 41.71, *p* < 0.001.

**TABLE 2:** Independent sample *t*-tests on stigma dimensions between study groups.

Variable	PLWHA (*N* = 300)		PLWHPT (*N* = 300)		*t*	*p*
	*M*	*SD*	*M*	*SD*		
Internalised stigma (INS)	44.53	8.82	64.60	2.81	37.56	0.00**
Perceived stigma (PSC)	43.29	6.82	60.57	2.22	41.71	0.00**
Enacted stigma (ENS)	11.28	1.81	9.00	0.00	-11.79	0.00**

*N*, number of participants; *SD*, standard deviation; *M*, mean; *t, t*-statistic; PLWHA, people living with HIV and/or AIDS; PLWHPT, people living with hypertension.

** , *p*-value is significant at 0.05 level (1-tail).

## Discussion

Results of the study revealed that PLWHA significantly experience more enacted HIV and AIDS stigma over other stigma dimensions relative to PLWHPT. This finding is consistent with other previous studies, where PLWHA reported higher incidence rates of stigma experiences relative to people with other chronic conditions (Blake et al. 2017; dos Santos et al. 2014; Hewko et al. 2018; Idemudia & Matemela 2012; Mak et al. 2007). This finding perhaps might not have been anticipated, considering the vigorous global state of the HIV and AIDS educational programmes that have already been put in place with the rationale of combating stigma. But the present study reveals that HIV and AIDS stigma still prevails. This implies that stigmatising tendencies cannot be simply inferred to lack of HIV and AIDS knowledge. This claim has been asserted by findings of the studies by Maughan-Brown (2010) and Wong (2013) who found that stigmatising attitudes were not associated with poor HIV knowledge, indicating that people still stigmatised PLWHA regardless of the adequate knowledge they have about the illness.

The implication of high enacted stigma experiences in this study can be explicated with reference to Nyblade et al’s. (2018) instrumental and symbolic stigma conceptualisation. From the instrumental stigma perspective, as instrumental stigmatisation occurs as a result of a perceived threat to an individual’s health that produces a negative attitude towards an individual who is perceived to be the threat, the present finding could imply that stigmatisers perceived that being around PLWHA could be a threat to their health, as a result, discriminating them to avoid contagion. From the symbolic stigma perspective, as symbolic stigma occurs as a result of the association made between the stigmatised individual with negative attitudes towards the associated stigmatised symbol (Nyblade et al. 2018). Therefore, PLWHA are stigmatised on a metaphorical level because of the symbols attached to the HIV illness. These are aspects such as prejudices associated with behaviours linked to HIV and/or AIDS transmission such as sexual immorality, promiscuity, homosexual sex, injecting drug use, sex work, etc., that precipitate HIV and/or AIDS stigma enactments. As it is documented that the common mode of HIV transmission in South Africa is mostly through heterosexual sex (UNAIDS & WHO 2013), the stigma enactments could be based on the sexual immorality associated with HIV and/or AIDS transmission. Understanding of immorality as the cause of HIV and AIDS enables the stigmatisers to distance themselves from sufferers (Grodensky et al. 2015), manifesting as enacted HIV and AIDS stigma, where the stigmatisers link sex to sin and sin to sex (Wangen 2010).

Research confirms this assertion where it has been found that if it is known that an individual has contracted HIV through socially deviant behaviours such as multiple sex partners, homosexual sex or injection drug practices, the individual is more stigmatised with more hostile reactions (Von Hippel, Brener & Horwit 2018). This notion is consistent with other research findings (Maulana, Krumeich & Van Den Borne 2009; Muoghalu & Jegede 2013). Experiences of internalised and perceived HIV and AIDS stigma were less reported in this population relative to PLWHPT. The lower experience of internalised and perceived HIV and AIDS may imply that participants could have accomplished a sense of self-acceptance, positive self-concept and increased self-compassion, which serve as protective factors against such stigma. This claim is supported by research findings (Block 2009; Emlet, Tozay & Raveis 2011). Fewer experiences of internalised and perceived stigma among PLWHA in this study is however in contrast with the results of a South African study by Gilbert and Walker (2009), where among their participants high levels of internalised and perceived HIV and AIDS stigma experiences were reported.

With regard to stigma among PLWHPT, significant experiences of internalised and perceived stigma relative to PLWHA were reported. This finding might be because of the commonly attributed lifestyle-related aetiological factors for hypertension, such as being overweight or obesity or heavy alcohol use (Maredza et al. 2016) to name a few. Therefore, PLWHPT might perceive themselves as personally responsible for their illness and anticipate stigmatisation from others as well. However, empirical evidence to support this claim is non-existent. Studies that assessed stigma among hypertensive patients is scarce; however, in a study conducted by Adams and Carter (2010), when compared to diabetes, hypertension was found to be relatively stigma-inducing.

## Recommendations and implications for future studies

Stigma reduction interventions are indicated to curb the adverse effects of stigma on people with these chronic illnesses. Specifically, additional knowledge in psychotherapeutic approaches for nurses will assist PLWHA and PLWHPT to cope with stigmatisation. Nurses can assist in counselling their patients (individual and group counselling), and sufficient care and support for these group of people will also assist in ameliorating stigmatisation. Also, because HIV and/or AIDS and hypertension are not contagious diseases, nurses should ensure that sufferers are given equal rights within the hospital settings while government should ensure that their rights within the society are guaranteed. These may be backed up with legal and policy interventions. Nurses can further assist in educating and creating additional awareness on these diseases. This will assist the patients in gaining more information about their status and how to better manage their conditions. Factors that still maintain HIV and/or AIDS stigma enactments internalised and perceived stigma among PLWHPT are worth further empirical investigation.

## Conclusion

Stigmatisation of PLWHA is still persistent in South African communities. Enacted HIV and AIDS stigma was the prevalent stigma type over other stigma dimensions among PLWHA. Stigma is existent among PLWHPT, and internalised and perceived stigma were common among hypertensive patients.
